# Association between visceral adiposity index and risk of diabetes and prediabetes: Results from the NHANES (1999–2018)

**DOI:** 10.1371/journal.pone.0299285

**Published:** 2024-04-25

**Authors:** Dongdong Zheng, Chuanxin Zhao, Kai Ma, Zhishen Ruan, Haoran Zhou, Haopeng Wu, Feng Lu

**Affiliations:** 1 Shandong University of Traditional Chinese Medicine, Jinan, Shandong, China; 2 Dongying People’s Hospital (Dongying Hospital of Shandong Provincial Hospital Group), Dongying, Shandong, China; 3 Affiliated Hospital of Shandong University of Traditional Chinese Medicine, Jinan, Shandong, China; Instituto Nacional de Geriatria, MEXICO

## Abstract

**Objective:**

To investigate the association between the visceral adiposity index and the prevalence of diabetes and prediabetes in the US adult population.

**Method:**

We conducted a cross-sectional study using data from the National Health and Nutrition Examination Survey (NHANES) from 1999 to 2018 for ten consecutive years, including 18745 eligible participants. The weighted multivariate logistic model and fitting curve were used to explore the correlation and dose-response relationship between visceral adiposity index (VAI) and diabetes (DM) and prediabetes in the general population and the prevalence of different subgroups.

**Results:**

In the fully adjusted continuous model, the risk of diabetes and prediabetes in the general population increased 0.15 times [1.15 (1.10,1.20), p<0.0001] with every increase of 1 unit of VAI. In the fully adjusted classification model, with the lowest quartile array Q1 of VAI as the reference group, Q2 of the second Quantile group, Q3 of the third Quantile group, and Q4 of the Quartile group increased 0.26 times [1.26 (1.10,1.44), p<0.001], 0.65 times [1.65 (1.43,1.89), p<0.0001], 1.60 times [2.60 (2.28,2.97), p<0.0001] respectively with the risk of diabetes and prediabetes. The above results showed that VAI was positively associated with the prevalence of diabetes and prediabetes, and the fitted curves showed a non-linear trend. (P for non-linear = 0<0.05). The results of the subgroup population were consistent with the total population and a significant interaction was found in gender (P for interaction<0.0001).

**Conclusion:**

In conclusion, we found a non-linear positive association between VAI and the risk of diabetes and prediabetes in the US adult population and found that women have a higher risk of diabetes and prediabetes than men; therefore, we should focus on the female population, and we call for the use of VAI to manage the development of diabetes and prediabetes in the clinical setting.

## Introduction

Some studies suggest that the prevalence of diabetes will increase to 783 million by 2025, and more and more people will die of diabetes and its complications [1]. Prediabetes is a high-risk state of diabetes, defined by the blood sugar variable that is higher than average but lower than the threshold of diabetes. Every year, 5–10% of patients with prediabetes will progress to diabetes; it is estimated that more than 470 million people will have prediabetes by 2030. Research shows that prediabetes increases the risk of kidney disease, neuropathy, retinopathy, and Great vessel disease [2]. We must also be aware of this disease state. Therefore, we should carry out prevention and treatment according to the pathological mechanism of diabetes and precursor diabetes to reduce the occurrence of clinical complications.

The visceral adiposity index is calculated from waist circumference, body mass index, triglyceride, and High-density lipoprotein cholesterol, which indirectly expresses visceral fat function, a new gender-specific index. Research has shown that VAI is an essential indicator of "visceral fat function" and insulin sensitivity, and its increase is closely related to the risk of cardiac metabolism [3]. Previous studies have demonstrated that VAI is a good predictor of the risk of metabolic syndrome [4], non-alcoholic fatty liver disease [5], chronic kidney disease [6], and cardiovascular disease [7]. This implies that VAI is also highly likely to predict the development of diabetes and prediabetes.

Previous VAI studies were primarily conducted in diabetes [8–10]. Little attention was paid to the pre-disease state of diabetes; prior studies have never been verified in the American population. Therefore, this article studies the correlation between visceral obesity index and diabetes and pre-disease diabetes in American adults.

## Method

### Study population

**Fig 1:**
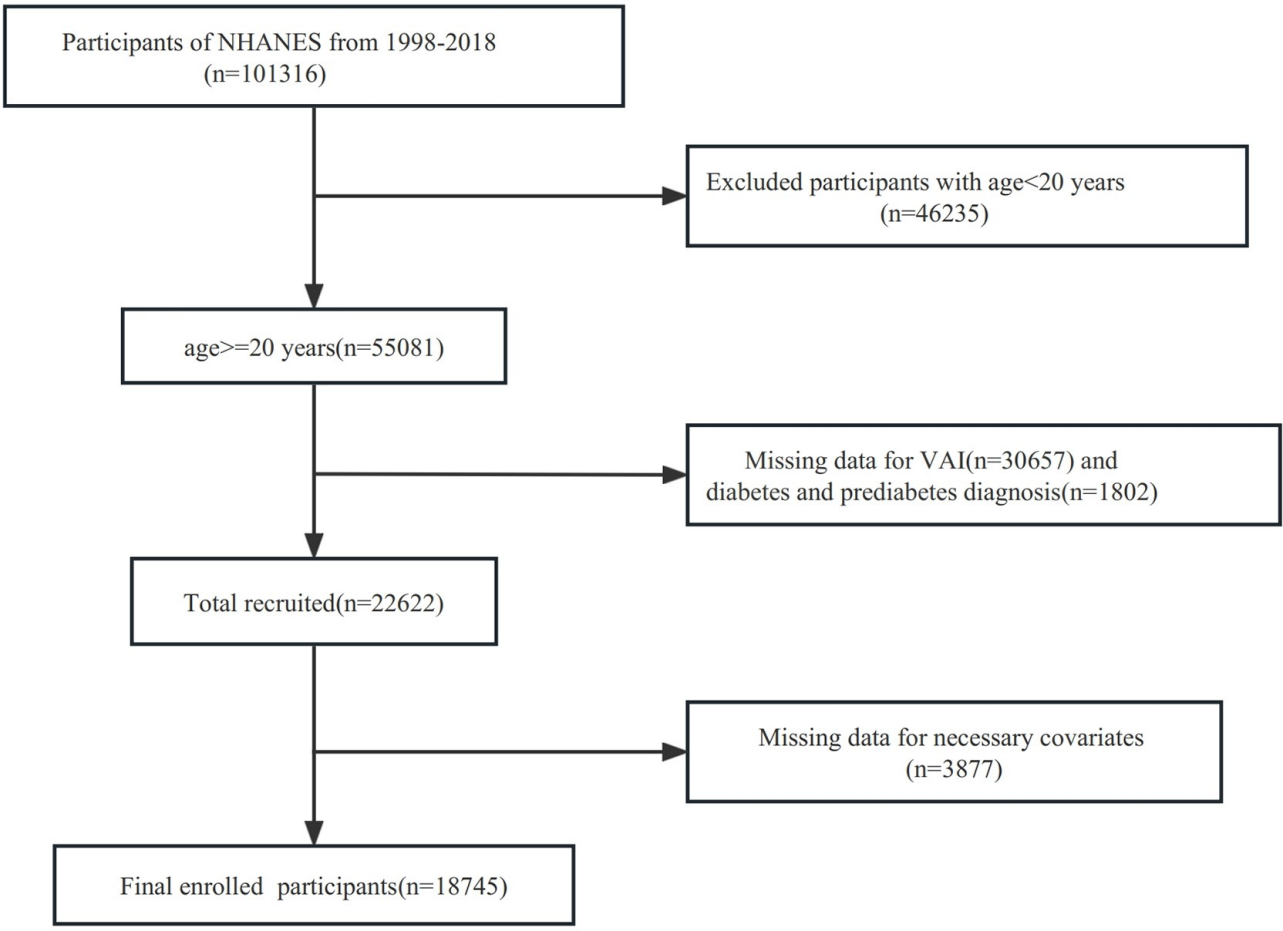
Flowchart of participants’ selection.

NHANES is a nationally representative cross-sectional study designed to assess the health and nutritional status of an ambulatory population in the US It was approved by the Research Ethics Review Board of the Centers for Disease Control and Prevention, and all participants provided written informed consent, so no further ethical review was required. Moreover, our study complied with the guidelines of the Epidemiologic Statement to enhance the reporting of observational studies [11]. We screened all participants aged 20 and above from the NHANES database from 1999 to 2018 (n = 55081). We excluded the lack of diabetes and pre-diagnosis of diabetes (n = 1802) and VAI data (n = 30657). Then, we excluded the lack of necessary covariates (n = 3877). Finally, an analysis was conducted on 18745 subjects. Further details are illustrated in **Fig 1**.

### Diagnosis of diabetes and prediabetes

According to the 2013 Diabetes Association Guidelines [12], diabetes can be defined to meet any of the following: 1) Self-reporting doctors diagnosed as diabetes; 2) Currently using hypoglycemic drugs or injecting insulin; 3) Random blood glucose ≥ 11.1 mmol/L; 4) Glycated hemoglobin level ≥ 6.5%; 5) fasting plasma glucose (FPG) level ≥ 7.0 mmol/L; 6) 2-hour OGTT blood glucose level ≥ 11.1 mmol/L. Prediabetes can be defined as meeting any of the following: 1) Self-reported doctors diagnosed as prediabetes; 2) 5.7% ≤ Glycated hemoglobin < 6.5%; 3) fasting plasma glucose (FPG) was between 5.6 mmol/L and 7.0 mmol/L; 4) The 2-hour OGTT blood glucose level ranges from 7.8 mmol/L to 11.1 mmol/L. In this analysis, diabetes and prediabetes were considered as the final event.

### Calculation of VAI

VAI is calculated using two independent equations for males and females. The formula for males is VAI = (WC/[39.68+1.88 × BMI)] × (TG/1.03) × (1.31/HDL), the procedure for women is VAI = [WC/(36.58+1.89 × BMI)] × (TG/0.81) × (1.52/HDL), where BMI represents body mass index in kg/m^2^, WC represents waist circumference in cm, TG represents triglyceride in mmol/L, and HDL represents High-density lipoprotein in mmol/L.

### Covariates

Covariates include age, gender (Male, Female), race/ethnicity (Non-Hispanic white, Non-Hispanic black, Mexican Americans, Others), education level (Less than high school, High school or equivalent, College or above), marital status (Married/living with partner, Divorced/widowed/separated, Never married), family poverty income ratio (PIR)(≤1.30, >1.30to≤3.50, PIR>3.50), smoking status (Former, Now, Never), alcohol user (Former, Mild/moderate, Heavy, Never), estimated glomerular filtration rate (eGFR, mL/min/1.73m^2^), fasting plasma glucose (FPG, mmol/L) and anti-hyperlipidemic drugs(yes/no). Hypertension is defined as any of the following: 1) self-reported diagnosis of hypertension by a doctor; 2) Currently using antihypertensive drugs; 3) The average value of blood pressure measured three times, with systolic blood pressure (SBP)>140mmHg or diastolic blood pressure (DBP)>90mmHg. Hyperlipidemia can be diagnosed by meeting any of the following conditions: 1) Diagnosed as hypertriglyceridemia (TG > = 150mg/dL or > = 1.70mmol/L); 2) Hypercholesterolemia can be diagnosed if any of the following conditions are met: 1. T.C. > = 200mg/dL (> = 5.18mmol/L) 2. LDL> = 130mg/dL (> = 3.37mmol/L) 3. Male HDL<40mg/dL (<1.04mmol/L), female HDL<50mg/dL (<1.30mmol/L); 3) Use lipid-lowering drugs. Cardiovascular diseases come from self-reported cardiovascular diseases diagnosed by doctors, including coronary heart disease, heart failure, heart attack, stroke, and angina pectoris. The family poverty income ratio (PIR) is an index for the ratio of family income to poverty, and it is calculated by dividing family income by the poverty threshold specific to the survey year and accounting for family size. PIR was characterized into three categories: low-income (PIR≤1.30), middle-income (PIR >1.30 to ≤3.50), and high-income (PIR>3.50) [13]. The formula for estimating eGFR is the Chronic Kidney Disease Epidemiology Collaboration (CKD-EPI) equation [14].

### Statistical analysis

This study conducted a statistical analysis using R (version 4.3.1). We included the weights, sdmvstra, and sdmvpsu variables in all analyses. The strata statement names the variables that form the strata. For the Continuous NHANES, the variable that identifies the sample strata is named sdmvstra.In NHANES, the variable representing the sample clusters is named sdmvpsu (masked PSUs) [15]. Continuous variables are represented as mean (SD). The Categorical variable is expressed as percentages with a 95% confidence interval. Weighted Student’s t-test, Mann Whitney U test, and Chi-squared test were used to compare the differences between the two groups. The statistical significance level was set as P<0.05.

The relationship between the VAI level and the risk of diabetes and prediabetes was explored through a multi-factor Logistic regression model. The OR and 95%CI are calculated in the "survey" R package. Model 1 did not adjust for any covariates. Model 2 adjusted for age, gender, race/ethnicity, education level, marital status, PIR, smoking status, and alcohol user. Model 3 further adjusted eGFR, hypertension, hyperlipidemia, cardiovascular disease, and anti-hyperlipidemic drugs. We divide the multi-factor Logistic regression model into a classification and continuous models. The VAI level is quartered in the classification model, and the non-linear trend is tested using each Quartile’s median as a constant variable. In addition, we investigated the association between VAI and fasting blood glucose.

In addition, we performed subgroup analysis in subgroups of age (<60,≥60), gender (Male/Female), race/ethnicity (Non-Hispanic white, Non-Hispanic black, Mexican Americans, Other), an education level (Less than high school, High school or equivalent, College or above), marital status (Married/living with partner, Divorced/widowed/separated, Never married), PIR (≤1.30, >1.30 to ≤3.50, >3.50), smoking status (Former, Now, Never), alcohol user (Former, Mild/moderate, Heavy, Never), eGFR (<60, ≥60), hypertension (Yes/No), hyperlipidemia (Yes/No), cardiovascular disease (Yes/No), and anti-hyperlipidemic drugs(Yes/No) to investigate the association between VAI levels and risk of diabetes and prediabetes. The level of statistical significance was set at p<0.05.

## Results

### Characteristics of the study population

As shown in **Tables 1** and **S1**, there were 18745 participants, including 9245 males, representing approximately 40165962 males, and 9500 females representing approximately 41146624 females nationwide. The mean (SD) age of the participants was 47.02 (0.24) years, and the mean (SD) for VAI was 2.13 (0.03). The prevalence of prediabetes was 39.01%, and the prevalence of diabetes was 18.43%. **Table 1** shows the characteristics of participants by quartiles of baseline VAI. All variables were statistically significant across the four VAI groups. Compared to participants in the Q1 group, participants in the Q4 group were typically male, older, non-Hispanic white, more educated, more widowed/divorced/separated participants, more current smokers and former drinkers, higher poverty-to-income ratios and lower eGFR, and more participants with hypertension, hyperlipidemia, and cardiovascular disease, as well as those taking lipid-lowering medications. **Table 2** shows the characteristics of participants by disease state. VAI mean (SD) was significantly higher with diabetes and prediabetes mean (SD) 2.51 (0.04) than without prediabetes mean (SD) 1.71 (0.03). As shown in **S2 Table**, we also found that VAI values were progressively higher in none prediabetes, prediabetes, and diabetes patients.

**Table 1:** Characteristics of study population divided by VAI Quartile.

Variable	Total	Q1(VAI≤0.92) (n = 4689)	Q2(0.92<VAI≤1.52)(n = 4686)	Q3(1.52<VAI≤2.57)(n = 4685)	Q4(VAI>2.57) (n = 4685)	P value
Age(years old)	47.02(0.24)	43.84(0.40)	46.59(0.35)	48.23(0.33)	49.60(0.28)	< 0.0001
Gender						0.03
Male	9245(49.32)	2477(51.24)	2311(47.98)	2230(48.19)	2227(50.14)	
Female	9500(50.68)	2212(48.76)	2375(52.02)	2455(51.81)	2458(49.86)	
Race/ethnicity						< 0.0001
Non-Hispanic White	71.09(66.41,75.76)	67.54(65.04,70.05)	71.14(68.77,73.52)	71.54(69.14,73.94)	74.33(71.90,76.76)	
Non-Hispanic Black	9.85 (8.93,10.77)	15.14(13.38,16.91)	10.81 (9.54,12.08)	8.14 (7.05, 9.23)	4.93 (4.19, 5.67)	
Mexican American	7.82 (6.88, 8.76)	6.30(5.27, 7.33)	7.03(5.84, 8.22)	8.67(7.35, 9.99)	9.41(8.06,10.76)	
Others	11.25(10.15,12.34)	11.01 (9.55,12.48)	11.02 (9.56,12.47)	11.65(10.22,13.09)	11.32 (9.56,13.09)	
Educational level						< 0.0001
Less than high school	16.35(15.21,17.49)	12.55(11.20,13.90)	15.10(13.62,16.58)	17.82(16.45,19.19)	20.22(18.76,21.67)	
High school or equivalent	24.01(22.34,25.68)	20.22(18.45,21.99)	24.18(22.61,25.75)	24.69(22.90,26.49)	27.17(25.05,29.29)	
College or above	59.64(56.71,62.57)	67.23(64.92,69.55)	60.73(58.55,62.91)	57.49(55.39,59.59)	52.61(50.29,54.93)	
Marital status						< 0.0001
Married/living with partner	65.50(62.07,68.94)	62.63(60.70,64.56)	64.79(62.77,66.80)	66.49(64.49,68.49)	68.31(66.31,70.31)	
Divorced/widowed/separated	17.86(16.75,18.97)	14.78(13.38,16.17)	18.23(16.94,19.53)	18.34(16.95,19.72)	20.25(18.68,21.82)	
Never married	16.64(15.66,17.62)	22.60(20.94,24.25)	16.98(15.29,18.67)	15.17(13.59,16.75)	11.44(10.08,12.80)	
PIR						< 0.0001
≤1.30	20.18(18.77,21.59)	17.67(16.09,19.25)	19.31(17.68,20.94)	20.65(18.97,22.33)	23.31(21.64,24.98)	
>1.30 to ≤3.50	36.24(34.32,38.16)	34.21(32.37,36.04)	35.04(32.97,37.11)	37.85(35.92,39.79)	38.03(36.10,39.95)	
>3.50	43.58(40.90,46.25)	48.13(45.86,50.39)	45.65(43.15,48.16)	41.49(39.13,43.85)	38.67(36.35,40.98)	
Smoking status						< 0.0001
Former	25.98(24.19,27.77)	23.64(21.68,25.60)	25.41(23.51,27.32)	26.28(24.46,28.09)	28.76(26.93,30.58)	
Now	21.30(19.87,22.73)	17.28(15.70,18.85)	21.02(19.31,22.73)	22.04(20.32,23.77)	25.11(23.55,26.67)	
Never	52.72(50.40,55.05)	59.09(56.92,61.25)	53.57(51.48,55.66)	51.68(49.54,53.82)	46.13(44.14,48.12)	
Alcohol user						< 0.0001
Former	14.54(13.33,15.76)	9.66 (8.46,10.85)	13.52(12.22,14.81)	15.91(14.51,17.30)	19.44(17.66,21.22)	
Mild/moderate	53.94(51.21,56.66)	61.13(59.18,63.09)	53.35(51.46,55.24)	52.07(49.93,54.22)	48.79(46.25,51.34)	
Heavy	20.87(19.76,21.98)	20.12(18.72,21.52)	22.40(20.64,24.16)	21.13(19.69,22.56)	19.79(18.15,21.43)	
Never	10.65 (9.55,11.75)	9.09 (8.02,10.16)	10.73 (9.33,12.13)	10.89 (9.43,12.36)	11.98(10.49,13.47)	
eGFR(mL/min/1.73m^2^)	94.86(0.31)	98.71(0.47)	95.17(0.44)	93.48(0.43)	91.83(0.43)	< 0.0001
FPG(mmol/L)	5.83(0.02)	5.45(0.02)	5.62(0.02)	5.87(0.03)	6.40(0.05)	< 0.0001
VAI	2.13(0.03)	0.64(0.00)	1.20(0.00)	1.98(0.01)	4.86(0.08)	< 0.0001
Hypertension						< 0.0001
Yes	37.17(35.17,39.18)	24.79(23.01,26.57)	34.89(32.80,36.99)	40.72(38.79,42.65)	49.14(47.20,51.07)	
No	62.83(59.99,65.67)	75.21(73.43,76.99)	65.11(63.01,67.20)	59.28(57.35,61.21)	50.86(48.93,52.80)	
Hyperlipidemia						< 0.0001
Yes	72.10(68.67,75.52)	42.57(40.81,44.32)	63.00(61.16,64.83)	85.09(83.76,86.41)	99.89(99.78,99.99)	
No	27.90(26.53,29.28)	57.43(55.68,59.19)	37.00(35.17,38.84)	14.91(13.59,16.24)	0.11 (0.01, 0.22)	
CVD						< 0.0001
Yes	8.58 (7.84, 9.31)	5.35 (4.51, 6.18)	7.22 (6.35, 8.08)	9.95 (8.93,10.97)	12.05(10.85,13.24)	
No	91.42(87.57,95.27)	94.65(93.82,95.49)	92.78(91.92,93.65)	90.05(89.03,91.07)	87.95(86.76,89.15)	
Glucose metabolism state						< 0.0001
None prediabetes	47.58(45.29,49.87)	61.20(59.45,62.95)	52.62(50.45,54.79)	43.85(41.74,45.95)	31.58(29.60,33.56)	
Prediabetes	38.45(36.38,40.52)	32.44(30.66,34.22)	37.19(35.23,39.14)	40.83(38.62,43.03)	43.76(41.87,45.64)	
Diabetes	13.97(13.01,14.93)	6.36 (5.57, 7.15)	10.19 (9.18,11.21)	15.33(13.83,16.82)	24.66(22.87,26.46)	
Anti-hyperlipidemic drugs						< 0.0001
Yes	16.62(15.46,17.78)	10.86 (9.57,12.15)	15.14(13.83,16.45)	18.88(17.11,20.64)	22.01(20.44,23.59)	
No	83.38(79.82,86.94)	89.14(87.85,90.43)	84.86(83.55,86.17)	81.12(79.36,82.89)	77.99(76.41,79.56)	

Abbreviation: fasting plasma glucose data were missing for 32 of 18745 participants. PIR: family poverty income ratio; FPG: fasting plasma glucose; VAI: visceral adiposity index; CVD: cardiovascular disease; eGFR: estimated glomerular filtration rate.

Continuous variables are represented as mean (SD). The Categorical variable is expressed as percentages with their 95% confidence interval.

**Table 2:** Characteristics of study population divided by different disease states.

Variable	Total	None prediabetes (n = 7978)	Diabetes and prediabetes (n = 10767)	P value
Age(years old)	47.02(0.24)	40.61(0.28)	52.83(0.24)	< 0.0001
Gender				< 0.0001
Male	49.40(47.12,51.67)	43.53(42.43,44.62)	54.72(53.67,55.78)	
Female	50.60(48.37,52.84)	56.47(55.38,57.57)	45.28(44.22,46.33)	
Race/ethnicity				< 0.001
Non-Hispanic White	71.09(66.41,75.76)	72.79(70.78,74.79)	69.54(67.18,71.91)	
Non-Hispanic Black	9.85 (8.93,10.77)	9.39(8.26,10.53)	10.26(9.06,11.46)	
Mexican American	7.82 (6.88, 8.76)	7.16(6.19,8.12)	8.42(7.17,9.68)	
Others	11.25(10.15,12.34)	10.66 (9.34,11.98)	11.78(10.55,13.00)	
Educational level				< 0.0001
Less than high school	16.35(15.21,17.49)	13.37(12.26,14.49)	19.05(17.87,20.23)	
High school or equivalent	24.01(22.34,25.68)	22.14(20.74,23.55)	25.70(24.29,27.11)	
College or above	59.64(56.71,62.57)	64.48(62.50,66.46)	55.25(53.38,57.11)	
PIR				0.01
≤1.30	20.18(18.77,21.59)	19.80(18.34,21.26)	20.53(19.24,21.83)	
>1.30to≤3.50	36.24(34.32,38.16)	35.13(33.45,36.81)	37.24(35.85,38.64)	
>3.50	43.58(40.90,46.25)	45.07(42.96,47.18)	42.22(40.34,44.10)	
Marital status				< 0.0001
Married/living with partner	65.50(62.07,68.94)	63.96(62.41,65.52)	66.90(65.53,68.27)	
Divorced/widowed/separated	17.86(16.75,18.97)	14.04(13.04,15.03)	21.33(20.18,22.48)	
Never married	16.64(15.66,17.62)	22.00(20.53,23.47)	11.77(10.69,12.85)	
PIR				0.01
≤1.30	20.18(18.77,21.59)	19.80(18.34,21.26)	20.53(19.24,21.83)	
>1.30 to ≤3.50	36.24(34.32,38.16)	35.13(33.45,36.81)	37.24(35.85,38.64)	
>3.50	43.58(40.90,46.25)	45.07(42.96,47.18)	42.22(40.34,44.10)	
Smoking status				< 0.0001
Former	25.98(24.19,27.77)	21.10(19.69,22.51)	30.40(28.99,31.82)	
Now	21.30(19.87,22.73)	22.71(21.11,24.31)	20.01(18.90,21.13)	
Never	52.72(50.40,55.05)	56.19(54.31,58.07)	49.58(48.05,51.11)	
Alcohol user				< 0.0001
Former	14.54(13.33,15.76)	11.51(10.40,12.62)	17.30(16.19,18.41)	
Mild/moderate	53.94(51.21,56.66)	54.90(52.90,56.91)	53.06(51.40,54.72)	
Heavy	20.87(19.76,21.98)	23.73(22.40,25.07)	18.27(17.19,19.36)	
Never	10.65 (9.55,11.75)	9.85 (8.38,11.33)	11.37(10.42,12.32)	
eGFR(mL/min/1.73m^2^)	94.86(0.31)	100.12(0.37)	90.09(0.34)	< 0.0001
FPG(mmol/L)	5.83(0.02)	5.08(0.01)	6.50(0.03)	< 0.0001
VAI	2.13(0.03)	1.71(0.03)	2.51(0.04)	< 0.0001
Hypertension				< 0.0001
Yes	37.17(35.17,39.18)	23.04(21.71,24.37)	50.00(48.64,51.36)	
No	62.83(59.99,65.67)	76.96(75.63,78.29)	50.00(48.64,51.36)	
Hyperlipidemia				< 0.0001
Yes	72.10(68.67,75.52)	61.97(60.51,63.43)	81.28(80.10,82.47)	
No	27.90(26.53,29.28)	38.03(36.57,39.49)	18.72(17.53,19.90)	
CVD				< 0.0001
Yes	8.58 (7.84, 9.31)	3.99 (3.48, 4.49)	12.74(11.87,13.61)	
No	91.42(87.57,95.27)	96.01(95.51,96.52)	87.26(86.39,88.13)	
Anti-hyperlipidemic drugs				< 0.0001
Yes	16.62(15.46,17.78)	6.54 (5.78, 7.29)	25.77(24.56,26.99)	
No	83.38(79.82,86.94)	93.46(92.71,94.22)	74.23(73.01,75.44)	

Abbreviation: fasting plasma glucose data were missing for 32 of 18745 participants. PIR: family poverty income ratio; FPG: fasting plasma glucose; VAI: visceral adiposity index; CVD: cardiovascular disease; eGFR: estimated glomerular filtration rate.

Continuous variables are represented as mean (SD). The Categorical variable is expressed as percentages with their 95% confidence interval.

### VAI level and diabetes and prediabetes

**Fig 2:**
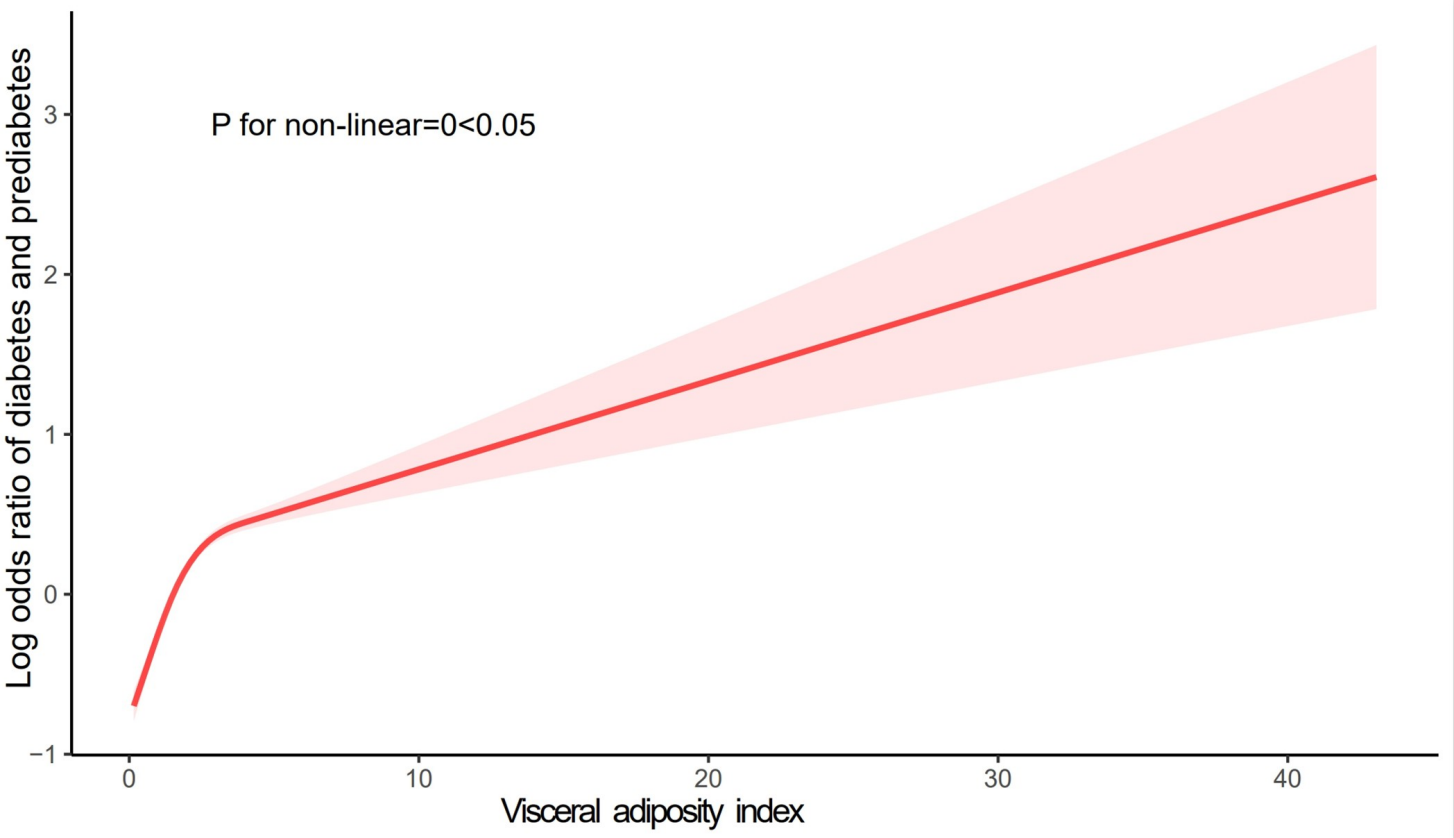
The association between VAI and the risk of diabetes and prediabetes. Age, gender, race/ethnicity, educational level, marital status, PIR, smoking status, alcohol user eGFR, hypertension, hyperlipidemia, CVD, and anti-hyperlipidemic drugs were adjusted.

As shown in **Table 3**, in the fully adjusted continuous model, the risk of diabetes and prediabetes in the total population increases 0.15 times [1.15 (1.10,1.20), p<0.0001] with every increase of 1 unit of VAI, and the results are statistically significant. In the fully adjusted classification model, taking the Quartile group Q1 with the lowest VAI as the reference group, the risk of diabetes and prediabetes in Q2, Q3, and Q4 and the general population increased by 0.26 times [1.26 (1.10,1.44), p<0.001], 0.65 times [1.43 (1.40,1.89), p<0.0001], 1.60 times [2.60 (2.28,2.97), p<0.0001] respectively. The above results indicated that VAI was positively associated with the risk of diabetes and prediabetes, and the fitted curves showed a non-linear trend (P for non-linear = 0<0.05). Further details are illustrated in **Fig 2**. In addition, we found a non-linear positive correlation between VAI and fasting plasma glucose, as shown in **S3 Table** and **S1 Fig**.

**Table 3:** According to the VAI of different models, the OR value and 95% CI of diabetes and prediabetes in all participants.

VAI	Event(%)	Diabetes and prediabetes OR (95%CI)					
		Model 1	P	Model 2	P	Model 3	P
Per 1 increment	10767(57.44%)	1.23(1.18,1.29)	<0.0001	1.20(1.15,1.26)	<0.0001	1.15(1.10,1.20)	<0.0001
Quartile							
Q1	2099 (38.80%)	1.00(reference)		1.00(reference)		1.00(reference)	
Q2	2477(47.38%)	1.42(1.27,1.59)	<0.0001	1.34(1.18,1.53)	<0.0001	1.26(1.10,1.44)	<0.001
Q3	2895(56.16%)	2.02(1.81,2.26)	<0.0001	1.86(1.63,2.12)	<0.0001	1.65(1.43,1.89)	<0.0001
Q4	3296(68.42%)	3.42(3.06,3.81)	<0.0001	3.11(2.75,3.53)	<0.0001	2.60(2.28,2.97)	<0.0001
p for trend			<0.0001		<0.0001		<0.0001

Model 1 was adjusted for none.

Model 2 was adjusted for age, gender, race/ethnicity, educational level, marital status, PIR, smoking status, and alcohol user.

Model 3 was adjusted for age, gender, race/ethnicity, educational level, marital status, PIR, smoking status, alcohol user eGFR, hypertension, hyperlipidemia, CVD, and anti-hyperlipidemic drugs.

Abbreviation: CI: Confidence interval.

### Subgroup analysis

As shown in **Table 4**, taking Group Q1 as a reference, Group Q2, Q3, and Q4 are positively correlated with the risk of diabetes and prediabetes, which is consistent with the research results of the general population. We found a significant interaction (p for interaction< 0.0001) in gender (male, female). In men, the risk of diabetes and prediabetes increased 1.06-fold in the Q4 group, using the Q1 group as a reference, and in women, the risk of diabetes and prediabetes increased 2.38-fold in the Q4 group, using the Q1 group as a reference.

**Table 4:** Results of subgroup analysis.

Character	Q1	Q2	Q3	Q4	P for trend	P for interaction
Age(years old)						0.75
<60	1.00(ref)	1.21(1.05,1.41)	1.56(1.33,1.82)	2.30(1.97,2.69)	<0.0001	
≥60	1.00(ref)	1.23(0.97,1.56)	1.58(1.21,2.07)	2.83(2.18,3.66)	<0.0001	
Gender						< 0.0001
Male	1.00(ref)	1.21(1.01,1.46)	1.40(1.14,1.71)	2.06(1.69,2.51)	<0.0001	
Female	1.00(ref)	1.35(1.13,1.61)	1.98(1.66,2.36)	3.38(2.81,4.07)	<0.0001	
Race/ethnicity						0.18
Non-Hispanic White	1.00(ref)	1.26(1.06,1.50)	1.61(1.34,1.94)	2.69(2.26,3.19)	<0.0001	
Non-Hispanic Black	1.00(ref)	1.38(1.13,1.68)	2.18(1.73,2.76)	2.39(1.77,3.22)	<0.0001	
Mexican American	1.00(ref)	1.06(0.77,1.46)	1.40(1.02,1.91)	2.11(1.47,3.03)	<0.0001	
Others	1.00(ref)	1.22(0.87,1.71)	1.58(1.13,2.22)	2.29(1.60,3.27)	<0.0001	
Educational level						0.88
Less than high school	1.00(ref)	1.24(0.94,1.63)	1.70(1.27,2.28)	2.96(2.15,4.09)	<0.0001	
High school or equivalent	1.00(ref)	1.36(1.03,1.78)	1.61(1.20,2.18)	2.55(1.87,3.46)	<0.0001	
College or above	1.00(ref)	1.23(1.04,1.46)	1.64(1.39,1.94)	2.50(2.08,3.01)	<0.0001	
Marital status						0.67
Married/living with partner	1.00(ref)	1.25(1.06,1.48)	1.56(1.31,1.86)	2.58(2.13,3.11)	<0.0001	
Divorced/widowed/separated	1.00(ref)	1.27(0.94,1.72)	1.63(1.21,2.21)	2.77(2.04,3.78)	<0.0001	
Never married	1.00(ref)	1.25(0.91,1.71)	1.96(1.42,2.70)	2.26(1.57,3.24)	<0.0001	
PIR						0.08
≤1.30	1.00(ref)	1.23(0.98,1.53)	1.84(1.45,2.33)	2.50(1.96,3.18)	<0.0001	
>1.30 to ≤3.50	1.00(ref)	1.30(1.05,1.62)	1.95(1.55,2.45)	2.61(2.06,3.30)	<0.0001	
>3.50	1.00(ref)	1.26(1.01,1.56)	1.39(1.14,1.70)	2.74(2.12,3.53)	<0.0001	
Smoking status						0.08
Former	1.00(ref)	1.17(0.91,1.50)	1.61(1.26,2.04)	2.93(2.15,4.00)	<0.0001	
Now	1.00(ref)	1.27(0.97,1.68)	2.14(1.52,3.03)	2.43(1.76,3.35)	<0.0001	
Never	1.00(ref)	1.28(1.11,1.49)	1.47(1.23,1.77)	2.51(2.08,3.03)	<0.0001	
Alcohol user						0.92
Former	1.00(ref)	1.21(0.85,1.72)	1.50(1.07,2.09)	2.38(1.59,3.56)	<0.0001	
Mild/moderate	1.00(ref)	1.35(1.13,1.61)	1.76(1.46,2.12)	2.66(2.19,3.24)	<0.0001	
Heavy	1.00(ref)	1.11(0.82,1.49)	1.50(1.09,2.05)	2.68(1.95,3.67)	<0.0001	
Never	1.00(ref)	1.15(0.80,1.66)	1.51(1.03,2.22)	2.18(1.42,3.35)	<0.001	
eGFR(mL/min/1.73m2)						0.87
≥60	1.00(ref)	1.26(1.10,1.45)	1.64(1.42,1.89)	2.61(2.27,3.01)	<0.0001	
<60	1.00(ref)	0.99(0.56,1.77)	1.65(0.86,3.16)	2.20(1.16,4.15)	0.003	
Hypertension						0.33
Yes	1.00(ref)	1.39(1.12,1.73)	1.95(1.53,2.49)	3.17(2.50,4.01)	<0.0001	
No	1.00(ref)	1.20(1.03,1.41)	1.50(1.28,1.77)	2.32(1.95,2.77)	<0.0001	
Hyperlipidemia						0.38
Yes	1.00(ref)	1.29(1.09,1.54)	1.59(1.33,1.88)	2.53(2.15,2.96)	<0.0001	
No	1.00(ref)	1.20(0.98, 1.46)	1.83(1.42, 2.35)	3.39(0.83,13.91)	<0.0001	
CVD						0.27
Yes	1.00(ref)	1.26(0.80,1.98)	2.20(1.40,3.45)	3.59(2.22,5.80)	<0.0001	
No	1.00(ref)	1.26(1.10,1.44)	1.61(1.40,1.85)	2.54(2.21,2.92)	<0.0001	
Anti-hyperlipidemic drugs						0.45
Yes	1.00(ref)	1.19(0.81,1.73)	1.30(0.88,1.91)	2.33(1.50,3.60)	<0.0001	
No	1.00(ref)	1.30(1.13,1.50)	1.80(1.57,2.07)	2.85(2.46,3.30)	<0.0001	

Abbreviation: PIR: family poverty income ratio; CVD: cardiovascular disease; eGFR: estimated glomerular filtration rate; ref: reference.

## Discussion

Here, we explored the correlation between VAI value and risk of diabetes and prediabetes in a nationally representative large sample cross-sectional study. In the US population, VAI values were nonlinearly and positively associated with the occurrence of diabetes and prediabetes, with subgroup analyses consistent with the results of the total population, and significant interactions were found in gender.

A cross-sectional study from Zhengzhou, China, investigated 5457 participants by stratified random sampling and observed that the VAI value positively correlated with prediabetes [16]. In Chinese adults aged 20–50, Liu et al. found that higher VAI values were positively associated with prediabetes and diabetes [17]. Aysha Alkhalaqi et al. studied a random sample of 1103 adult Qatari nationals and long-term residents over the age of 20 years in Qatar. They found not only that VAI was a stronger predictor of T2DM compared to body obesity index (VAI) and BMI but also that the association between VAI and T2DM was more robust in females than in males [8]. A prospective Cohort study (ATTICA study) from Greece with 10-year follow-up randomly recruited 1514 participants and found that VAI significantly increased the risk of diabetes by 22% (OR = 1.22; 95% CI: 1.09, 1.37), indicating that VAI may be a valuable indicator for predicting the long-term development of diabetes [9]. In conclusion, this study further verified the correlation between VAI and diabetes and prediabetes in American adults.

Some studies have shown that obesity is the leading risk factor for diabetes [18–21], especially Abdominal obesity [22], whose etiology involves the interaction between genetic, environmental, physiological, behavioral, social, and economic factors [23]. Although BMI is the most commonly used method for evaluating obesity, an essential limitation of using BMI is that it cannot reflect actual body fat [24]. Waist circumference (WC) is a simple and reliable measurement method for Abdominal obesity, which has been used to measure total fat [25]. Still, it cannot distinguish between subcutaneous and visceral fat [26]. Some studies have shown that compared with subcutaneous fat, visceral fat will produce more free fatty acids, thus increasing the risk of insulin resistance and diabetes [27, 28]. VAI includes anthropometric measurements (BMI and WC) and metabolic variables (TG and HDL), which can provide more information about visceral adipose tissue and is therefore considered a substitute for visceral fat.

In addition, our study showed that VAI predicted the risk of diabetes and prediabetes and found that women had a higher risk of developing diabetes and prediabetes than men, which may be related not only to aging [29, 30] but also to the depletion of estrogen [31, 32]. In addition, we investigated the relationship between VAI and fasting plasma glucose, which is in keeping with the findings of Qin Y et al. [33].

The mechanism of action of VAI in prediabetes and diabetes is unclear, and studies have shown that it is not only related to the fact that high levels of TG may decrease the amount and the activity of insulin reactors on adipocytes, but also to the fact that lower levels of HDL may lead to decreased insulin secretion and sensitivity [34]. In addition, dyslipidemia can cause insulin resistance (IR) through induction of inflammation, endoplasmic network stress, and lipid toxicity [35], all of which are strongly associated with the development of diabetes and prediabetes.

### Limitations

Our research also has several limitations. First, since the current study is cross-sectional, we cannot draw a causal conclusion from our results. Although we adjusted various confounding factors in the model, we cannot exclude the influence of other unmeasured potential confounding factors, such as dietary intake and environmental exposure. Finally, results might not be extrapolated to other countries because of national differences.

## Conclusion

In conclusion, we found that VAI values were positively and nonlinearly associated with the development of diabetes and prediabetes in the US adult population. In subgroup analyses, we found a significant gender interaction, with women having a higher risk of diabetes and prediabetes than men. Therefore, we should be more concerned about the risk of diabetes and prediabetes in the female population, and further research is needed to explore the mechanism of VAI and the development of diabetes and prediabetes. Therefore, we should pay more attention to the risk of diabetes and prediabetes in the female population, and further research is needed to explore the mechanism of VAI and diabetes and prediabetes. We call for the use of VAI to manage the development of diabetes and prediabetes in the clinical setting.

## Supporting information

S1 Fig Association between VAI and fasting plasma glucose. Age, gender, race/ethnicity, educational level, marital status, PIR, smoking status, alcohol user, eGFR, hypertension, hyperlipidemia, CVD and anti-hyperlipidemic drug were adjusted.
(TIF)

S1 Table Characteristics of study population divided by VAI Quartile. (DOCX)

S2 Table Characteristics of study population divided by different disease states. (DOCX)

S3 Table Relationship between VAI and fasting plasma glucose in different models. (DOCX)

S1 Data (XLSX)
