# Supplementary figures and images for: Association between visceral adiposity index and risk of diabetes and prediabetes: Results from the NHANES (1999–2018)
Source: PLoS One. 2024 Apr 25;19(4):e0299285. doi: 10.1371/journal.pone.0299285 (PMC11045124; doi:10.1371/journal.pone.0299285)

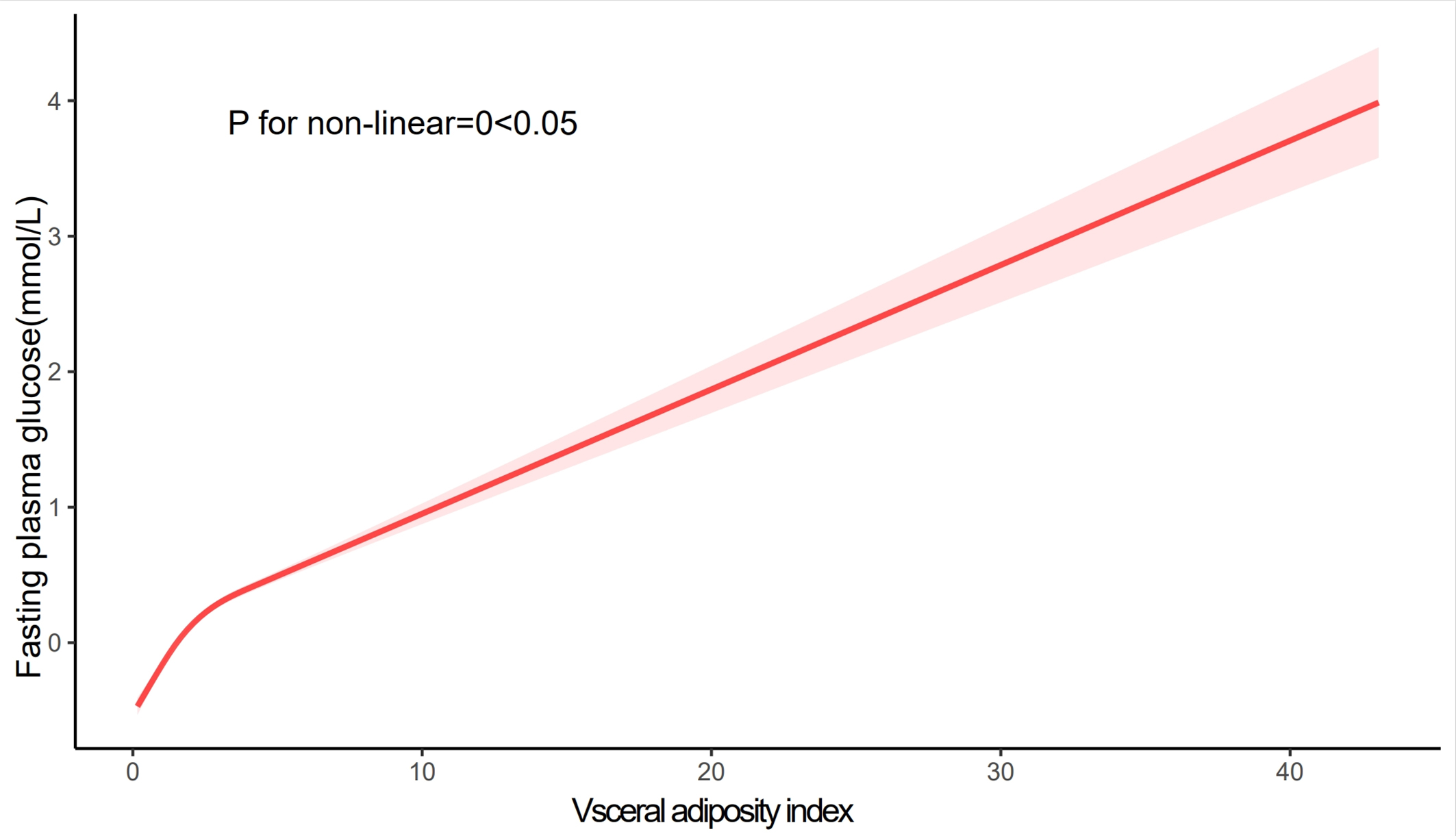

Supplement: S1 Fig — Age, gender, race/ethnicity, educational level, marital status, PIR, smoking status, alcohol user, eGFR, hypertension, hyperlipidemia, CVD and anti-hyperlipidemic drug were adjusted. (TIF) [file pone.0299285.s001.tif]
