# Supplementary material for: Association between visceral adiposity index and risk of diabetes and prediabetes: Results from the NHANES (1999–2018)
Source: PLoS One. 2024 Apr 25;19(4):e0299285. doi: 10.1371/journal.pone.0299285 (PMC11045124; doi:10.1371/journal.pone.0299285)
Supplement: S1 Table — (DOCX) [file pone.0299285.s002.docx]

**S1 Table** Characteristics of study population divided by VAI Quartile

| Variable | Total | Q1(VAI≤0.92)  (n=4689) | Q2(0.92<VAI≤1.52)(n=4686) | Q3(1.52<VAI≤2.57)(n=4685) | Q4(VAI>2.57)  (n=4685) | P value |
| --- | --- | --- | --- | --- | --- | --- |
| Age(years old) | 47.02(0.24) | 43.84(0.40) | 46.59(0.35) | 48.23(0.33) | 49.60(0.28) | < 0.0001 |
| Gender |  |  |  |  |  | 0.03 |
| Female | 41146624.72(50.60) | 10205521.40(48.76) | 10771385.52(52.02) | 10360183.72(51.81) | 9809534.09(49.86) |  |
| Male | 40165962.36(49.40) | 10726203.72(51.24) | 9935493.46(47.98) | 9637842.00(48.19) | 9866423.19(50.14) |  |
| Race/ethnicity |  |  |  |  |  | < 0.0001 |
| Non-Hispanic White | 57802041.66(71.09) | 14138285.34(67.54) | 14731541.09(71.14) | 14306551.59(71.54) | 14625663.64(74.33) |  |
| Non-Hispanic Black | 8006435.66( 9.85) | 3169836.88(15.14) | 2238698.77(10.81) | 1627605.45( 8.14) | 970294.57( 4.93) |  |
| Mexican American | 6359017.98( 7.82) | 1318141.05(6.30) | 1455456.38(7.03) | 1733496.31(8.67) | 1851924.24(9.41) |  |
| Others | 9145091.78(11.25) | 2305461.84(11.01) | 2281182.74(11.02) | 2330372.37(11.65) | 2228074.83(11.32) |  |
| Educational level |  |  |  |  |  | < 0.0001 |
| Less than high school | 13294442.84(16.35) | 2626650.13(12.55) | 3125881.68(15.10) | 3564056.63(17.82) | 3977854.40(20.22) |  |
| High school or equivalent | 19522472.50(24.01) | 4232022.44(20.22) | 5006192.31(24.18) | 4937867.41(24.69) | 5346390.34(27.17) |  |
| College or above | 48495671.75(59.64) | 14073052.55(67.23) | 12574804.98(60.73) | 11496101.68(57.49) | 10351712.54(52.61) |  |
| Marital status |  |  |  |  |  | < 0.0001 |
| Married/living with partner | 53262567.91(65.50) | 13109169.49(62.63) | 13415493.49(64.79) | 13297317.61(66.49) | 13440587.33(68.31) |  |
| Divorced/widowed/separated | 14520232.56(17.86) | 3092742.46(14.78) | 3775791.17(18.23) | 3667603.17(18.34) | 3984095.76(20.25) |  |
| Never married | 13529786.60(16.64) | 4729813.17(22.60) | 3515594.31(16.98) | 3033104.94(15.17) | 2251274.18(11.44) |  |
| PIR |  |  |  |  |  | < 0.0001 |
| ≤1.30 | 16412318.63(20.18) | 3697688.45(17.67) | 3998385.93(19.31) | 4130390.09(20.65) | 4585854.16(23.31) |  |
| >1.30 to ≤.50 | 29466927.97(36.24) | 7159849.49(34.21) | 7255628.45(35.04) | 7569615.66(37.85) | 7481834.37(38.03) |  |
| >3.50 | 35433340.48(43.58) | 10074187.17(48.13) | 9452864.60(45.65) | 8298019.96(41.49) | 7608268.75(38.67) |  |
| Smoking status |  |  |  |  |  | < 0.0001 |
| Former | 21123008.89(25.98) | 4947849.35(23.64) | 5262124.66(25.41) | 5254882.28(26.28) | 5658152.60(28.76) |  |
| Now | 17317905.27(21.30) | 3616362.63(17.28) | 4351983.45(21.02) | 4408339.15(22.04) | 4941220.05(25.11) |  |
| Never | 42871672.91(52.72) | 12367513.13(59.09) | 11092770.87(53.57) | 10334804.29(51.68) | 9076584.62(46.13) |  |
|  |  |  |  |  |  |  |
| Alcohol user |  |  |  |  |  | < 0.0001 |
| Former | 11826343.11(14.54) | 2021358.93( 9.66) | 2798968.17(13.52) | 3180840.25(15.91) | 3825175.76(19.44) |  |
| Mild/moderate | 43857345.47(53.94) | 12795903.32(61.13) | 11047247.96(53.35) | 10413839.05(52.07) | 9600355.15(48.79) |  |
| Heavy | 16969325.30(20.87) | 4212250.10(20.12) | 4638319.15(22.40) | 4225129.50(21.13) | 3893626.54(19.79) |  |
| Never | 8659573.20(10.65) | 1902212.76( 9.09) | 2222343.69(10.73) | 2178216.93(10.89) | 2356799.83(11.98) |  |
| eGFR(mL/min/1.73m^2^) | 94.86(0.31) | 98.71(0.47) | 95.17(0.44) | 93.48(0.43) | 91.83(0.43) | < 0.0001 |
| FPG(mmol/L) | 5.83(0.02) | 5.45(0.02) | 5.62(0.02) | 5.87(0.03) | 6.40(0.05) | < 0.0001 |
| VAI | 2.13(0.03) | 0.64(0.00) | 1.20(0.00) | 1.98(0.01) | 4.86(0.08) | < 0.0001 |
| Hypertension |  |  |  |  |  | < 0.0001 |
| Yes | 30225146.93(37.17) | 5188921.22(24.79) | 7225611.54(34.89) | 8142673.39(40.72) | 9667940.78(49.14) |  |
| No | 51087440.15(62.83) | 15742803.90(75.21) | 13481267.44(65.11) | 11855352.33(59.28) | 10008016.49(50.86) |  |
| Hyperlipidemia |  |  |  |  |  | < 0.0001 |
| Yes | 58622967.12(72.10) | 8909739.33(42.57) | 13044378.53(63.00) | 17015478.89(85.09) | 19653370.36(99.89) |  |
| No | 22689619.96(27.90) | 12021985.78(57.43) | 7662500.44(37.00) | 2982546.83(14.91) | 22586.91( 0.11) |  |
| CVD |  |  |  |  |  | < 0.0001 |
| Yes | 6973593.62( 8.58) | 1118872.33( 5.35) | 1494453.48( 7.22) | 1989907.59( 9.95) | 2370360.22(12.05) |  |
| No | 74338993.46(91.42) | 19812852.78(94.65) | 19212425.50(92.78) | 18008118.12(90.05) | 17305597.06(87.95) |  |
| Glucose metabolism state |  |  |  |  |  | < 0.0001 |
| None prediabetes | 38687968.11(47.58) | 12810347.58(61.20) | 10895791.99(52.62) | 8768395.82(43.85) | 6213432.71(31.58) |  |
| Prediabetes | 31265220.44(38.45) | 6790656.31(32.44) | 7700518.15(37.19) | 8164367.66(40.83) | 8609678.31(43.76) |  |
| Diabetes | 11359398.53(13.97) | 1330721.22( 6.36) | 2110568.83(10.19) | 3065262.23(15.33) | 4852846.25(24.66) |  |
| Anti-hyperlipidemic drugs |  |  |  |  |  | < 0.0001 |
| Yes | 13515056.65(16.62) | 2272661.73(10.86) | 3135358.03(15.14) | 3775445.22(18.88) | 4331591.68(22.01) |  |
| No | 67797530.43(83.38) | 18659063.39(89.14) | 17571520.95(84.86) | 16222580.50(81.12) | 15344365.59(77.99) |  |

Abbreviation: fasting plasma glucose data were missing for 32 of 18745 participants. PIR: family poverty income ratio; FPG: fasting plasma glucose; VAI: visceral adiposity index; CVD: cardiovascular disease; eGFR: estimated glomerular filtration rate.

Continuous variables are represented as mean (SD). The Categorical variable is expressed as the expanded frequency (percentage)
