# Supplementary material for: Association between visceral adiposity index and risk of diabetes and prediabetes: Results from the NHANES (1999–2018)
Source: PLoS One. 2024 Apr 25;19(4):e0299285. doi: 10.1371/journal.pone.0299285 (PMC11045124; doi:10.1371/journal.pone.0299285)
Supplement: S2 Table — (DOCX) [file pone.0299285.s003.docx]

**S2 Table** Characteristics of study population divided by different disease states

| variable | total | None prediabetes  (n=7978) | Prediabetes  (n=7313) | Diabetes  (n=3454) | P value |
| --- | --- | --- | --- | --- | --- |
| Age(years old) | 47.02(0.24) | 40.61(0.28) | 50.61(0.28) | 58.96(0.33) | < 0.0001 |
| Gender |  |  |  |  | < 0.0001 |
| Male | 9245(49.32) | 3362(43.53) | 4056(55.89) | 1827(51.53) |  |
| Female | 9500(50.68) | 4616(56.47) | 3257(44.11) | 1627(48.47) |  |
| Race/ethnicity |  |  |  |  | < 0.0001 |
| Non-Hispanic White | 8872(47.33) | 4062(72.79) | 3406(70.65) | 1404(66.51) |  |
| Non-Hispanic Black | 3579(19.09) | 1424( 9.39) | 1372( 9.40) | 783(12.62) |  |
| Mexican American | 3286(17.53) | 1320(7.16) | 1287(8.35) | 679(8.62) |  |
| Others | 3008(16.05) | 1172(10.66) | 1248(11.60) | 588(12.25) |  |
| Educational level |  |  |  |  | < 0.0001 |
| Less than high school | 4772(25.46) | 1653(13.37) | 1916(17.44) | 1203(23.47) |  |
| High school or equivalent | 4327(23.08) | 1744(22.14) | 1745(25.00) | 838(27.64) |  |
| College or above | 9646(51.46) | 4581(64.48) | 3652(57.56) | 1413(48.89) |  |
| Marital status |  |  |  |  | < 0.0001 |
| Married/living with partner | 11618(61.98) | 4865(63.96) | 4625(67.36) | 2128(65.63) |  |
| Divorced/widowed/separated | 4001(21.34) | 1264(14.04) | 1693(19.60) | 1044(26.07) |  |
| Never married | 3126(16.68) | 1849(22.00) | 995(13.03) | 282( 8.30) |  |
| PIR |  |  |  |  | < 0.0001 |
| ≤1.30 | 5532(29.51) | 2227(19.80) | 2164(19.59) | 1141(23.14) |  |
| >1.30 to ≤3.50 | 7198(38.4) | 2977(35.13) | 2772(35.72) | 1449(41.44) |  |
| >3.50 | 6015(32.09) | 2774(45.07) | 2377(44.69) | 864(35.42) |  |
| Smoking status |  |  |  |  | < 0.0001 |
| Former | 4855(25.9) | 1639(21.10) | 2034(29.03) | 1182(34.18) |  |
| Now | 3908(20.85) | 1767(22.71) | 1554(20.99) | 587(17.34) |  |
| Never | 9982(53.25) | 4572(56.19) | 3725(49.98) | 1685(48.48) |  |
| Alcohol user |  |  |  |  | < 0.0001 |
| Former | 3299(17.6) | 1087(11.51) | 1280(15.00) | 932(23.63) |  |
| Mild/moderate | 9139(48.75) | 4029(54.90) | 3664(55.31) | 1446(46.85) |  |
| Heavy | 3751(20.01) | 1851(23.73) | 1424(19.83) | 476(13.98) |  |
| Never | 2556(13.64) | 1011( 9.85) | 945( 9.86) | 600(15.53) |  |
| eGFR(mL/min/1.73m^2^) | 94.86(0.31) | 100.12(0.37) | 92.09(0.37) | 84.58(0.54) | < 0.0001 |
| FPG(mmol/L) | 5.83(0.02) | 5.08(0.01) | 5.82(0.01) | 8.41(0.08) | < 0.0001 |
| VAI | 2.13(0.03) | 1.71(0.03) | 2.27(0.04) | 3.18(0.10) | < 0.0001 |
| Hypertension |  |  |  |  | < 0.0001 |
| Yes | 7813(41.68) | 1985(23.04) | 3378(43.11) | 2450(68.96) |  |
| No | 10932(58.32) | 5993(76.96) | 3935(56.89) | 1004(31.04) |  |
| Hyperlipidemia |  |  |  |  | < 0.0001 |
| Yes | 13780(73.51) | 5043(61.97) | 5708(78.57) | 3029(88.76) |  |
| No | 4965(26.49) | 2935(38.03) | 1605(21.43) | 425(11.24) |  |
| CVD |  |  |  |  | < 0.0001 |
| Yes | 2003(10.69) | 398( 3.99) | 773( 9.20) | 832(22.50) |  |
| No | 16742(89.31) | 7580(96.01) | 6540(90.80) | 2622(77.50) |  |
| Anti-hyperlipidemic drugs |  |  |  |  | < 0.0001 |
| Yes | 3353(17.89) | 499( 6.54) | 1309(18.29) | 1545(46.39) |  |
| No | 15392(82.11) | 7479(93.46) | 6004(81.71) | 1909(53.61) |  |

Abbreviation: fasting plasma glucose data were missing for 32 of 18745 participants. PIR: family poverty income ratio; FPG: fasting plasma glucose; VAI: visceral adiposity index; CVD: cardiovascular disease; eGFR: estimated glomerular filtration rate.

Continuous variables are represented as mean (SD). The Categorical variable is expressed as percentages with their 95% confidence interval.
