# Supplementary material for: Association between visceral adiposity index and risk of diabetes and prediabetes: Results from the NHANES (1999–2018)
Source: PLoS One. 2024 Apr 25;19(4):e0299285. doi: 10.1371/journal.pone.0299285 (PMC11045124; doi:10.1371/journal.pone.0299285)
Supplement: S3 Table — (DOCX) [file pone.0299285.s004.docx]

**S3 Table** Relationship between VAI and fasting plasma glucose in different models

| Exposure | Model 1 | | Model 2 | | Model 3 | |
| --- | --- | --- | --- | --- | --- | --- |
|  | β(95%CI) | P value | β(95%CI) | P value | β(95%CI) | P value |
| VAI | 0.14(0.11,0.17) | <0.0001 | 0.13( 0.10, 0.16) | <0.0001 | 0.12( 0.09, 0.15) | <0.0001 |
| VAI（Quartile） | | | | | | |
| Q1 | 1.00(reference) |  | 1.00(reference) |  | 1.00(reference) |  |
| Q2 | 0.17(0.12,0.22) | <0.0001 | 0.12( 0.07, 0.18) | <0.0001 | 0.11( 0.06, 0.16) | <0.0001 |
| Q3 | 0.42(0.35,0.49) | <0.0001 | 0.33( 0.27, 0.40) | <0.0001 | 0.31( 0.23, 0.38) | <0.0001 |
| Q4 | 0.96(0.86,1.05) | <0.0001 | 0.84( 0.74, 0.93) | <0.0001 | 0.79( 0.69, 0.88) | <0.0001 |
| p for trend |  | <0.0001 |  | <0.0001 |  | <0.0001 |

Model 1 was adjusted for none.

Model 2 was adjusted for age, gender, race/ethnicity, educational level, marital status, PIR, smoking status, and alcohol user.

Model 3 was adjusted for age, gender, race/ethnicity, educational level, marital status, PIR, smoking status, alcohol user, eGFR, hypertension, hyperlipidemia, CVD, and anti-hyperlipidemic drugs.

Abbreviation: CI: Confidence interval.
